# Outcomes after attempted external cephalic version with two prior cesarean deliveries

**DOI:** 10.1002/pmf2.70465

**Published:** 2026-09-25

**Authors:** Emily Schlussel Markovic, Itamar Futterman, Joselle O'Brien, Nelli Fisher, Tirtza Spiegel Strauss, Howard Minkoff, Rodney McLaren

**Affiliations:** ^1^ Obstetrics and Gynecology Maimonides Medical Center Brooklyn New York USA

**Keywords:** blood transfusion, breech, cesarean delivery, external cephalic version, malpresentation, NICU admission, trial of labor after cesarean

## Abstract

**Objective:**

American college of obstetricians and gynecologists (ACOG) considers patients with two prior cesarean deliveries (CDs) to be candidates for a trial of labor and separately recommends that external cephalic version (ECV) be offered for malpresentation. However, little is known about the safety, success rate, or effect on maternal and neonatal outcomes of ECV among patients with two prior CDs. This study aimed to evaluate obstetric outcomes after ECV in patients with two prior CDs to guide counseling.

**Study design:**

This was a retrospective cohort study of patients with two prior CDs with non‐anomalous, singleton births after 36 weeks’ gestation utilizing the US Natality Database from 2018 to 2023. Pregnant patients with breech pregnancies who had an ECV attempt were compared with those who did not. The adverse maternal outcomes included blood transfusion, uterine rupture, unplanned hysterectomy, and intensive care unit (ICU) admission. Adverse neonatal outcomes included 5‐minute Apgar score < 7, immediate or prolonged assisted ventilation, neonatal ICU (NICU) admission, seizure, and death. ECV success rate and mode of delivery were also assessed. Univariable and multivariable analyses were performed.

**Results:**

Among the 16,293 included births, 15,965 (98.0%) had a repeat CD (rCD) for malpresentation without an ECV attempt and 328 (2.0%) had an ECV attempt. Of these 328 ECVs, 223 (68.0%) were successful. Adverse maternal composite outcomes did not differ between births after an ECV attempt and those without (1.8% vs. 1.5%; adjusted odds ratio [aOR], 1.20; 95% confidence interval [CI], 0.53–2.71). Those who had an ECV attempt had a lower risk of adverse neonatal composite outcomes compared to those who did not (9.5% vs. 18.2%; aOR, 0.54; 95% CI, 0.37–0.79). Of the 223 who had a successful ECV, 152 (68.2%) underwent rCD without trial of labor after cesarean (TOLAC) and 71 (31.8%) underwent TOLAC. Among those with a successful ECV, there was no difference in adverse maternal or neonatal morbidities between those who had a TOLAC after successful ECV and those who did not.

**Conclusion:**

These data suggest that among patients with two prior CDs, an ECV had a success rate similar to the published rate in the general obstetric population and may be associated with decreased neonatal morbidity compared to patients with no attempted ECV.

## 1 INTRODUCTION

Four percent of term pregnancies present as breech at birth [1]. Breech vaginal delivery has fallen out of favor since the Term Breech Trial was published in 2000. That multicenter randomized controlled trial found a significantly higher risk for perinatal morbidity and mortality after intended vaginal delivery compared with intended cesarean delivery (CD) [2]. Although follow‐up of the original cohort revealed a lessened advantage among those undergoing cesarean section, rates of vaginal breech never rose to earlier rates [3]. Currently, most fetuses in breech presentation at term are delivered by CD [4, 5].

As CD confers maternal, neonatal, and future pregnancy risks [6], offering an external cephalic version (ECV) is recommended by all major societies [7]. ECV has been shown to reduce the proportion of non‐cephalic presentations at term [8]. Prior studies have demonstrated mixed results regarding the success of ECV with one prior CD [9, 10] with some studies suggesting a decreased success rate and an increased risk of adverse maternal and neonatal outcomes [9] compared to an ECV in an unscarred uterus. Other studies found no increase in adverse outcomes with an ECV after one prior CD [11]. However, limited data are available to estimate the effect of two prior CDs on ECV outcomes. This study aimed to evaluate the success rate of ECV as well as the rates of maternal and neonatal morbidity among patients with two prior CDs undergoing ECV.

## 2 MATERIALS AND METHODS

This was a retrospective cohort study of non‐anomalous, singleton births after 36 weeks’ gestation among patients with two prior CDs utilizing the US Natality Database from 2018 to 2023. Births were excluded from analysis if delivery was prior to 36 weeks’ gestation, prenatal care was initiated after 13 weeks’ gestation, there was a maternal history of less than or more than two prior CDs, or data were missing. Early initiation of prenatal care was used to ensure accurate pregnancy dating. This study was reviewed and determined to be exempt by the Institutional Review Board.

Two groups of births were compared: Those with two prior CDs who had an ECV attempt, and those who did not have an ECV attempt. Maternal and labor characteristics were compared between groups, including maternal age, parity, number of prior vaginal deliveries, body mass index (BMI) at delivery, gestational age at delivery, birth weight, and fetal presentation at delivery.

The primary outcomes were rates of composite maternal and neonatal adverse events. Adverse maternal outcomes included blood transfusion, uterine rupture, unplanned hysterectomy, and intensive care unit (ICU) admission. Adverse neonatal outcomes included 5‐minute Apgar less than seven, immediate or prolonged assisted ventilation, neonatal ICU (NICU) admission, seizure, and death. The secondary outcomes were the ECV success rate and mode of delivery. We also performed a subgroup analysis to compare maternal and neonatal morbidity among three subgroups of those with an ECV attempt: those who had a successful ECV and trial of labor after cesarean (TOLAC) resulting in vaginal delivery after cesarean (VBAC), those who had a successful ECV and TOLAC resulting in a repeat CD (rCD), and those who had a failed ECV and did not TOLAC. Only 3.5% of patients with breech presentation and two prior CDs underwent a TOLAC. Therefore, there was insufficient data to analyze this group separately.

Sample size calculations were based on the published literature [9] that assumed a 4% risk of uterine rupture in a scarred uterus and 0.7% in an unscarred uterus, as well as a 3% prevalence of breech presentation at term. Those assumptions required 3 years of national data to meet the minimum number of subjects for a study of patients undergoing ECV after one prior CD [9]. Given the expected lower prevalence of ECV among those with two prior CDs, we assumed we would need double the number of births in order to have an adequate number of subjects. Therefore, 6 years of data were collected.

To examine maternal and neonatal morbidity, adverse maternal and neonatal composite variables were compared between those who underwent an ECV attempt and those who did not undergo an ECV attempt. ECV success rate and mode of delivery after ECV attempt were also assessed.

Univariable and multivariable analyses were performed and adjustments were made for age, prior vaginal deliveries, BMI, and gestational age at delivery. Student's *t*‐test was used for continuous variables and Chi‐square test was used with categorical variables. A *p* value of <0.05 was considered significant. Odds ratios (ORs) were estimated with 95% confidence intervals (CIs). The main exposure of risk was an ECV prior to delivery. All analyses were performed using STATA 18.5, StataCorp.

## 3 RESULTS

There were 22,129,980 births in the United States between 2018 and 2023. After excluding births with delivery prior to 36 weeks’ gestation, first prenatal visit after 13 weeks, a maternal history of less than or more than two prior CDs, and those with missing data, there remained 502,679 multiparous pregnant people with singleton, non‐anomalous births. Among these 502,679 births, 16,293 (3.2%) had a breech presentation close to delivery and comprised our study population. This accounts for 3.2% of these births and is consistent with prior literature describing rates of breech presentation at term [12]. Of the 16,293 births, 15,965 (98.0%) had an rCD for malpresentation without a trial of labor or ECV attempt and 328 (2.0%) had an ECV attempt.

Demographic characteristics of those who underwent ECV attempt and those who did not are presented in Table 1. Those with an ECV attempt were younger (32.5 vs. 33.0 years; *p *= 0.029), had more prior vaginal deliveries (1 [interquartile range, IQR, 1–2] vs. 1 [IQR, 1–1], *p *< 0.001), lower BMI (29.9 ± 7.8 vs. 31.4 ± 9.3 kg/m^2^, *p *= 0.004), and later gestational age at delivery (39 weeks [IQR, 38–40] vs. 39 weeks [IQR, 37–39], *p *< 0.001). Birth weight did not differ between the groups (3363 vs. 3341 g, *p *= 0.481).

**TABLE 1:** Demographics.

	ECV attempted prior to delivery *N* = 328	Repeat CS and malposition with 2 prior CD without ECV attempt *N* = 15,965	*p* value
Maternal age (years)	32.5 ± 5.0	33.0 ± 4.9	**0.029**
Parity (prior live births)	3 (3, 4)	3 (3, 3)	**<0.001**
Prior vaginal deliveries (calculated variable)	1 (1, 2)	1 (1, 1)	**<0.001**
BMI at delivery (kg/m^2^)	29.9 ± 7.8	31.4 ± 9.3	**0.004**
GA at delivery (weeks)	39 (38, 40)	39 (37, 39)	**<0.001**
Birth weight (g)	3363.8 ± 499.6	3341.8 ± 560.9	0.481
Vertex presentation at birth	264 (80.5%)	–	–

*Note*: Data are presented as number (percent); continuous variables including age, BMI, and birth weight are presented as median ± interquartile range.

Abbreviations: BMI, body mass index; ECV, external cephalic version; GA, gestational age.

Bold value statistically significant *p* < 0.05.

The primary outcomes, maternal and neonatal morbidities, are shown in Tables 2 and 3, respectively. There was no evidence of increased maternal morbidity in either individual or composite adverse outcomes after an ECV attempt, as compared to those without an ECV attempt (1.8% vs. 1.5%; aOR, 1.20; 95% CI, 0.53–2.71). Those who had an ECV attempt had a lower risk of adverse neonatal composite compared to those who did not (9.5% vs. 18.2%; aOR 0.54; 95% CI, 0.37–0.79), driven by differences in immediate assisted ventilation (OR, 0.52; 95% CI, 0.32–0.84) and NICU admission (OR, 0.43; 95% CI, 0.27–0.68). There were no reports of uterine rupture among those who underwent an ECV attempt.

**TABLE 2:** Maternal outcomes.

	ECV attempted prior to delivery *N* = 328	Repeat CS and malposition with 2 prior CS without ECV attempt *N* = 15,965	Odds ratio, 95% CI
Composite maternal morbidity	6 (1.8%)	245 (1.5%)	1.20 (0.53–2.71)
Blood transfusion	4 (1.2%)	166 (1.0%)	1.17 (0.43–3.19)
Uterine rupture	0	25 (0.2%)	–
Unplanned peripartum hysterectomy	1 (0.3%)	54 (0.3%)	0.90 (0.12–6.53)
ICU admission	2 (0.6%)	53 (0.3%)	1.84 (0.45–7.59)

*Note*: Data are presented as *N* (%). Univariable analyses were performed due to low event outcomes.

Abbreviations: CI, confidence interval; ECV, external cephalic version; ICU, intensive care unit.

**TABLE 3:** Neonatal outcomes.

	ECV attempted prior to delivery *N* = 328	Repeat CS and malposition with 2 prior CS without ECV attempt *N* = 15,965	Odds ratio, 95% CI	Adjusted odds ratio,^a^ 95% CI
Composite neonatal morbidity	31 (9.5%)	2909 (18.2%)	**0.47 (0.32–0.68)**	**0.54 (0.37–0.79)**
5‐minute Apgar < 7	4 (1.2%)	400 (2.5%)	0.48 (0.18–1.29)	–
Immediate assisted ventilation	18 (5.5%)	1609 (10.1%)	**0.52 (0.32–0.84)**	–
Assisted ventilation > 6 h	6 (1.8%)	422 (2.6%)	0.69 (0.30–1.55)	–
NICU admission	19 (5.8%)	2013 (12.6%)	**0.43 (0.27–0.68)**	–
Neonatal seizure	0 (0%)	5 (0.03%)	–	–
Neonatal death	1 (0.3%)	20 (0.1%)	2.44 (0.33–18.22)	–

*Note*: Data are presented as *N* (%).

Abbreviations: CI, confidence interval; ECV, external cephalic version; NICU, neonatal intensive care unit.

^a^ Adjusted for maternal age, prior vaginal deliveries, body mass index, and gestational age at delivery.

**FIGURE 1:**
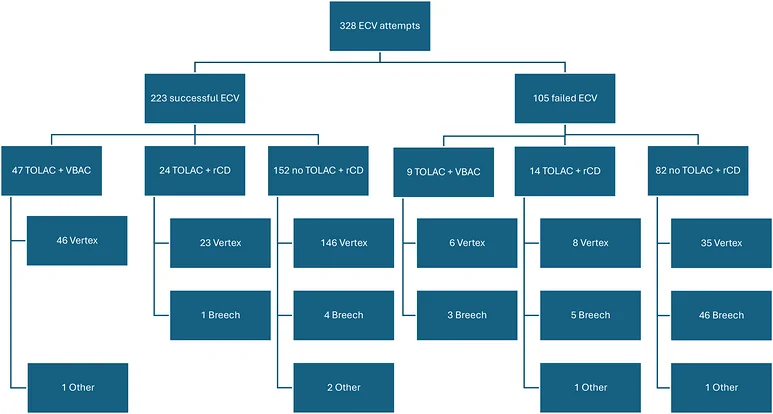
Mode of delivery and final fetal presentation of the ECV attempt group. ECV, external cephalic version; rCD, repeat cesarean delivery; TOLAC, trial of labor after cesarean; VBAC, vaginal birth after cesarean; Other, non‐vertex, non‐breech presentation.

The secondary outcomes, including success rates of ECV, are summarized in Figure 1, which also demonstrates the mode of delivery and final fetal presentation of the ECV attempt group. Of the 328 births with an ECV attempt, 223 (68.0%) were successful but eight reverted to malpresentation prior to delivery. Among the 215 cephalic deliveries after successful ECV, 146 (68.0%) had an rCD without TOLAC. Patients underwent an rCD without TOLAC had fewer prior vaginal deliveries than those who underwent a TOLAC (*n* = 69) (rCD = 1, [IQR, 1–1]; TOLAC = 2, [IQR, 1–3]; *p *< 0.001). Of the 105 patients with a failed ECV, 49 (46.7%) subsequently spontaneously verted and were vertex at birth and 3 (2.9%) had a breech vaginal delivery. Among the 223 successful ECVs, 71 (31.8%) had a trial of labor and among the 105 failed ECVs, 23 (21.9%) had a trial of labor. Among these combined 94 patients with a trial of labor, 52 (55.3%) had a spontaneous VBAC, 4 (4.3%) had an operative VBAC, and 38 (40.4%) underwent rCD in labor.

After noting that a low proportion of those with a successful ECV underwent a TOLAC, we performed an unplanned subgroup analysis to determine if those who underwent TOLAC after successful ECV were different from those who did not. We found no difference in individual or composite adverse maternal or neonatal morbidities between those who had a TOLAC after successful ECV and those who did not. Those who underwent a TOLAC were older (33.8 ± 4.9 vs. 31.8 ± 4.8, *p *= 0.004), had more prior vaginal deliveries (2 [IQR, 1–3] vs. 1 [IQR, 1–1], *p *< 0.001), and had lower BMI (26 [IQR, 23.1–30.4] vs. 28.7 [IQR, 24.4–34.8], *p *= 0.016). There was no difference in gestational age at delivery or birth weight.

A planned subgroup analysis (Tables 4 and 5) was then performed comparing the following three groups: those who had a successful ECV and TOLAC resulting in vaginal delivery, those who had a successful ECV and TOLAC resulting in an rCD, and those who had a failed ECV and did not TOLAC. There were 167 patients included in this subgroup analysis: 47 (28.1%) patients with a successful ECV and vaginal delivery, 24 (14.4%) patients with a successful ECV and rCD after TOLAC, and 96 patients (57.5%) with a failed ECV and rCD. There was no difference in maternal age, prior vaginal deliveries, BMI, gestational age at birth, or birth weight between these subgroups. There was also no difference in adverse maternal or neonatal morbidities.

**TABLE 4:** Demographics of subgroup analysis.

	Successful ECV + VBAC *N* = 47	Successful ECV + rCD in labor *N* = 24	Failed ECV + RCS *N* = 96	*p* value
Maternal age (years)	34.2 ± 4.6	33.0 ± 5.6	32.5 ± 5.2	0.171^a^
Parity (prior live births)	4 (3, 5)	3 (3, 4)	3 (3, 4)	0.001^b^
Prior vaginal delivery	2 (1, 3)	1 (1, 2)	1 (1, 2)	0.001^b^
BMI at delivery (kg/m^2^)	26 (22.4, 31.5)	26.1 (23.5, 29.0)	30.4 (25.2, 35.4)	0.018^b^
GA at delivery (weeks)	39 (38, 40)	39 (38, 40)	39 (38, 40)	0.933^b^
Birth weight (g)	3388.8 ± 577.2	3252.5 ± 439.7	3394.2 ± 498.4	0.394^a^

Abbreviations: BMI, body mass index; ECV, external cephalic version; GA, gestational age; VBAC, vaginal delivery after cesarean.

^a^ One‐way ANOVA.

^b^ Kruskal–Wallis test.

**TABLE 5:** Outcomes of subgroup analysis.

	Successful ECV + VBAC *N* = 47	Successful ECV + RCS in labor *N* = 24	OR, 95% CI^a^	Failed ECV + RCS *N* = 96	OR, 95% CI^a^
Adverse maternal composite	0 (0%)	2 (8.3%)	–	3 (3.1%)	–
Blood transfusion	0 (0%)	2 (8.3%)	–	1 (1.0%)	–
Uterine rupture	0 (0%)	0 (0%)	–	0 (0%)	–
Unplanned peripartum hysterectomy	0 (0%)	0 (0%)	–	1 (1.0%)	–
ICU admission	0 (0%)	0 (0%)	–	2 (2.1%)	–
Adverse neonatal composite	5 (10.6%)	2 (8.3%)	0.76 (0.14–4.26)	14 (14.6%)	1.43 (0.48–4.25)
5‐minute Apgar score < 7	2 (4.3%)	0 (0%)	–	0 (0%)	–
Immediate assisted ventilation	3 (6.4%)	2 (8.3%)	1.33 (0.21–8.57)	8 (8.3%)	1.33 (0.34–5.28)
Assisted ventilation use > 6 h	0 (0%)	1 (4.2%)	–	2 (2.1%)	–
NICU admission	1 (2.1%)	1 (4.2%)	2.00 (0.12–33.4)	11 (11.5%)	5.95 (0.74–47.57)
Neonatal seizures	0 (0%)	0 (0%)	–	0 (0%)	–
Neonatal death	1 (2.1%)	0 (0%)	–	0 (0%)	–

Abbreviations: CI, confidence interval; ECV, external cephalic version; GA, gestational age; ICU, intensive care unit; NICU, neonatal intensive care unit; OR, odds ratio; VBAC, vaginal delivery after cesarean.

^a^ Univariable analyses performed due to low event outcomes.

When considering vaginal delivery rate after ECV, 47 patients (21.1%) had a vaginal delivery after a successful ECV, and 9 patients (8.6%) had a vaginal delivery after a failed ECV.

## 4 DISCUSSION

This study demonstrated that patients with two prior CDs who underwent an external cephalic version, had no increased maternal morbidity and had decreased neonatal morbidity compared to those who underwent an elective rCD without attempting ECV. Additionally, among pregnant patients with a history of two prior CDs who had an ECV attempt, 68.0% had a successful ECV, similar to the rate reported among women undergoing version with no prior CDs [12]. Despite this high success rate, 68.0% of those with cephalic presentation after ECV subsequently had an rCD without TOLAC. While our data source did not allow us to survey patients and providers to understand why many successful ECVs occurred without subsequent TOLAC, it is possible that patients and providers would have allowed spontaneous labor but would not induce labor in a patient with two prior CDs due to risk of uterine rupture.

There is limited literature examining ECV after two prior cesarean sections. However, there have been several studies assessing ECV after a single CD and a high success rate has been reported. A large study in Spain calculated a 78.6% success rate, and a complication rate of 9.5% (including 7.1% with non‐reassuring fetal heart rates). Of those who attempted TOLAC after ECV, 80.8% achieved a VBAC [13]. These findings have been replicated in various global cohorts. A 2017 publication reported a 74% success rate after one prior CD without an increased complication rate compared to those without a uterine scar [14]. A 2021 systematic review similarly found that ECV after one previous CD had comparable success and risk to those without a previous CD. In the nine studies they reviewed, complications included abnormal fetal heart rate/cardiotocography and transient vaginal bleeding but no cases of uterine rupture [15]. Similarly, a retrospective study of 100 women who underwent ECV after prior CD had no cases of uterine rupture [16].

Other studies have examined outcomes of ECV after prior CD in different cohorts and assess the effect of obesity on ECV success and complication rate among patients with one prior CD. McLaren et al. found that there was no correlation between BMI and successful ECV or adverse outcomes among women with one prior CD [17].

A population‐based cohort study utilizing the US Natality Database demonstrated an increase in TOLAC rates from 14.4% in 2010 to 19.6% 2019 with a corresponding increase in successful VBAC over that time interval [18]. In our large cohort, we found that among women with two prior CDs. TOLAC occurred in 71 (31.8%) of patients who had a successful ECV after two prior CD with 47 (66.2%) achieving VBAC.

Our study had several strengths. This was a large contemporaneous, obstetrical cohort.

By including all births in the US Natality Database, we had a large enough cohort to identify 328 patients with two prior CDs who underwent an external cephalic version. This allowed us to assess serious adverse outcomes that have a relatively low prevalence. The diverse cohort also improves the generalizability of our findings. Finally, we performed a real‐world analysis which evaluated patients who underwent an ECV attempt rather than solely evaluating outcomes of those with a successful ECV and had a cephalic fetus at delivery since the ECV outcome is unknown to patients and providers at the time of clinical decision making.

A limitation of this study is that data are derived from a large administrative database which may include data entry errors and miscoding. We attempted to minimize such errors by excluding incomplete records from analyses. A further limitation was that analyses were limited to the variables that were available in the original dataset. Therefore, we were unable to account for some possible confounders. We did adjust for maternal age, prior vaginal deliveries, BMI, and gestational age at delivery. It is possible that there is a systemic bias whereby one group or the other differed in regard to some of the things we could not measure, for example, amniotic fluid quantity or placental location. We were also unable to ascertain the incidence placenta abruption, a known complication of ECV. However, there was no difference in maternal blood transfusion, which could serve as a surrogate for significant placental abruption.

## 5 CONCLUSION

In conclusion, our findings demonstrate that an ECV in patients with two prior CDs had a similar success rate to the general obstetric population and may be associated with decreased neonatal morbidity without additional maternal morbidity. This suggests that among patients with two prior CDs and malpresentation, an ECV may be a safe procedure that allows for TOLAC. These findings can inform patient counseling and may be of importance to those who strongly desire to avoid rCD. Future research should further include prospective studies to assess the safety and utility of ECV and TOLAC after two prior CDs.

## AUTHOR CONTRIBUTIONS

**Itamar Futterman**: Writing–review and editing. **Rodney Mclaren**: Supervision; conceptualization; writing–review and editing; formal analysis. **Tirtza Spiegel Strauss**: Writing–review and editing. **Nelli Fisher**: Writing–review and editing. **Emily Schlussel Markovic**: Investigation; writing–original draft; writing–review and editing; project administration. **Howard Minkoff**: Writing–review and editing; conceptualization. **Joselle O'Brien**: Writing–review and editing.

## CONFLICT OF INTEREST STATEMENT

The authors declare no conflicts of interest.

## FUNDING INFORMATION

The authors received no specific funding for this work.

## Data Availability

Data are available upon reasonable request.
